# A Mitochondria-Targeted Heptamethine Indocyanine Small Molecular Chelator for Attenuating Uranium Nephrotoxicity

**DOI:** 10.3390/ph17080995

**Published:** 2024-07-27

**Authors:** Zaizhi Du, Xie Huang, Zifei Wu, Mingquan Gao, Rong Li, Shenglin Luo

**Affiliations:** State Key Laboratory of Trauma and Chemical Poisoning, Institute of Combined Injury, Chongqing Engineering Research Center for Nanomedicine, College of Preventive Medicine, Third Military Medical University (Army Medical University), Chongqing 400038, China; duzaizhi@163.com (Z.D.); lirongyl@sina.com (R.L.)

**Keywords:** heptamethine, uranium decorporation, gallic acid, mitochondrion protection

## Abstract

Radionuclide uranium has both a chemical and radioactive toxicity, leading to severe nephrotoxicity as it predominantly deposits itself in the kidneys after entering into human bodies. It crosses renal cell membranes, accumulates in mitochondria and causes mitochondrial oxidative damage and dysfunction. In this study, a mitochondria-targeted heptamethine indocyanine small molecule chelator modified with gallic acid (IR-82) is synthesized for uranium detoxication. Both gallic acid and sulfonic acid, as two hydrophilic endings, make IR-82, being excreted feasibly through kidneys. Gallic acid with polyphenol groups has a steady metal chelation effect and potent antioxidant ability, which may facilitate IR-82-alleviated uranium nephrotoxicity simultaneously by enhancing uranium decorporation from the kidneys and reducing mitochondrial oxidative damage. Cell viability assays demonstrate that IR-82 can significantly improve the cell viability of uranium-exposed human renal (HK-2) cells. It is also demonstrated to accumulate in mitochondria and reduce mitochondrial ROS and total intracellular ROS, as well as intracellular uranium content. In vivo imaging experiments in mice show that IR-82 could be excreted out through kidneys. ICP-MS tests further reveal that IR-82 can efficiently decrease the uranium deposition in mouse kidneys. IR-82 treatment improves the animal survival rate and renal function of experimental mice after high-dose uranium exposure. Collectively, our study may evidence that the development of uranium decorporation agents with kidney–mitochondrion dual targeting abilities is a promising strategy for attenuating uranium-induced nephrotoxicity.

## 1. Introduction

With the extensive use of radionuclide uranium in the nuclear industry and military activities, the risk of uranium contamination is sharply increasing. Once radionuclide uranium is accidentally released into the environment, it will quickly enter into the biological system [1]. Due to its heavy metal chemical toxicity and radioactivity, uranium is a great threat to human health [2]. The biokinetic study shows that uranium tends to be deposited in the kidneys as uranyl ions (UO_2_^2+^) 24 h after contamination [3]. The heavy metal crosses cell membranes and the renal proximal tubules and is retained in subcellular organelles, such as mitochondria and lysosomes [4,5]. Mitochondria are cellular energy factories that produce adenosine triphosphate (ATP) through a process of oxidative phosphorylation, providing energy and maintaining the redox homeostasis of cells [6]. Excess ROS production can be induced after uranium overexposure in mitochondria. As a chemical and radioactive toxin, uranium severely inhibits the cytochrome C oxidase and mitochondrial respiratory chain, causing ROS production, mitochondrial dysfunction and apoptosis-mediated programmed cell death [7,8,9].

The decorporation of uranium and the elimination of excess ROS are considered as two main approaches to the mitigation of uranium-induced injury. On one hand, the decorporation approach prevents deposition in target organs, speeds up excretion, and thus lowers the overall buildup of uranium in tissue by using a specialized chelator to bind uranium and create stable complexes in vivo [10,11]. For examples, sodium bicarbonate (NaHCO_3_) is listed by the World Health Organization (WHO) as a decorporation drug for responding to radiation and nuclear emergencies [12]. ZnNa_3_-DTPA and CaNa_3_-DTPA are two uranium decorporation drugs approved by the United States Food and Drug Administration (FDA) [13]. Many compounds have been synthesized and tested for chelating uranium, but most exhibit a poor tissue specificity and high toxicity, precluding clinical application. On the other hand, a lot of antioxidants, such as glutathione (GSH), catechins (EGCG) and gallic acid (GA), have been found effectiveness in mitigating uranium-induced injury by scavenging intracellular ROS [14,15,16,17]. In its mechanism, uranium toxicity is found to be closely related to mitochondrial damage because uranium, like other heavy metals, can induce excess ROS and mitochondria-mediated apoptosis [18]. Bearing in mind that mitochondria play critical roles not only as an energy factory but also as the main source of endogenous ROS, mitochondria-targeting antioxidants can remove ROS more efficiently and offer a better therapeutic effect by protecting mitochondria [8,19]. Based on the two approaches above, we thus come to formulate a hypothesis that a chelator with kidney–mitochondrion dual-targeting abilities could be effective in attenuating uranium-induced nephrotoxicity.

In previous studies, we [20,21,22] and others [23,24,25] synthesized and reported a class of heptamethine indocyanine dyes with excellent mitochondria targeting. The structure–activity relationship studies indicate that a heptamethine core with a lipophilic cationic property is essential for mitochondrial targeting [26,27]. We developed a mitochondria-targeting radioprotectant (CY-TMP1) by further conjugating a potent ROS scavenger 2,2,6,6-tetramethylpiperidinyloxy (TEMPO), which was found to relieve radiation injury and accelerate wound repair [28]. As aforementioned, uranium is a mitochondrial toxin by causing excess ROS through both chemical and radioactive means. Therefore, in this work, we aim to design and report a new chelator for the mitigation of uranium-induced nephrotoxicity, based on a mitochondria-targeted heptamethine indocyanine small molecule. A new mitochondria-targeted heptamethine indocyanine small molecule chelator modified with GA is designed and synthesized (IR-82) for uranium detoxication. With two hydrophilic groups of sulfonic acid and GA, IR-82 exhibits water solubility and can be predominantly excreted through the kidneys. Its metabolic characteristic increases the chance for GA to chelate the uranium deposited in the kidneys. More importantly, GA, also as a potent antioxidant, efficiently scavenges uranium-induced Mito-ROS and mitigates mitochondrial oxidative damage after being taken into the mitochondria. Both the in vitro and in vivo preliminary results indicate that IR-82 with kidney–mitochondrion dual-targeting abilities may be a prospective strategy to promoting uranium decorporation and attenuating nephrotoxicity.

## 2. Results and Discussion

### 2.1. Synthesis and Structural Characterization

**Scheme 1:**
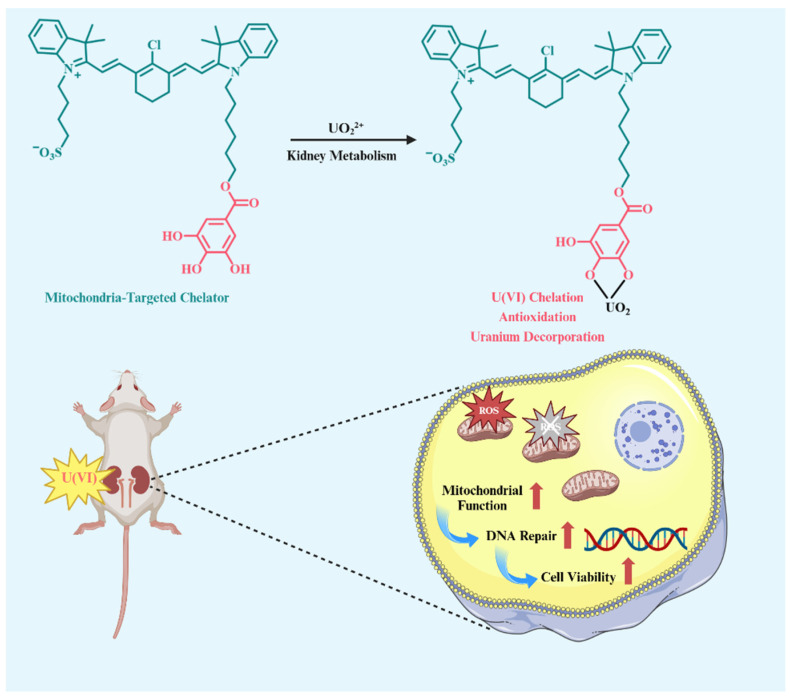
Chemical and biological mechanisms of a mitochondria-targeted heptamethine indocyanine small molecular chelator for attenuating the nephrotoxicity of uranium (VI). Gallic acid with polyphenol groups (red color) has a steady metal chelation effect and potent antioxidant ability, alleviating uranium nephrotoxicity simultaneously by enhancing uranium decorporation from the kidneys and reducing mitochondrial oxidative damage.

**Scheme 2:**
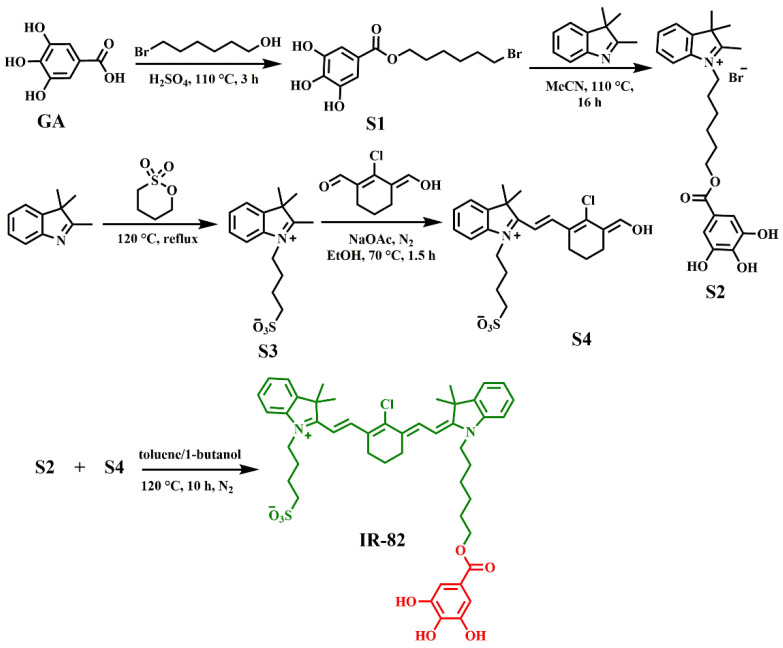
Synthetic route of a new mitochondria-targeted heptamethine indocyanine small molecule chelator (IR-82), starting with GA.

Considering the steady chelating ability of catechol groups to uranoyl ions, we used gallic acid (GA) as the starting material to synthesize a new mitochondria-targeted heptamethine indocyanine small molecular chelator IR-82, as shown in Scheme 1. The detailed synthetic route of IR-82 is presented in Scheme 2. Basically, using GA as a raw material, the key intermediates S1 and S2 were synthesized through esterification and *N*-substitution reactions, respectively. Meanwhile, the intermediate S3 and semi-cyanine intermediate S4 were synthesized according to our previous method [26]. Finally, by a condensation reaction between S2 and S4, IR-82 was obtained as a green powder. The structures of the key intermediates S1 and S2 were confirmed by ^1^H NMR. The structure of IR-82 was confirmed by ^1^H NMR, ^13^C NMR and high-resolution mass spectrometry (HRMS) (Figures S1–S5, Supplementary Materials). The detailed structural characterization data can be found in Materials and Methods.

### 2.2. Good Chelating Ability Towards UO2^2+^ in Water Solution

**Figure 1:**
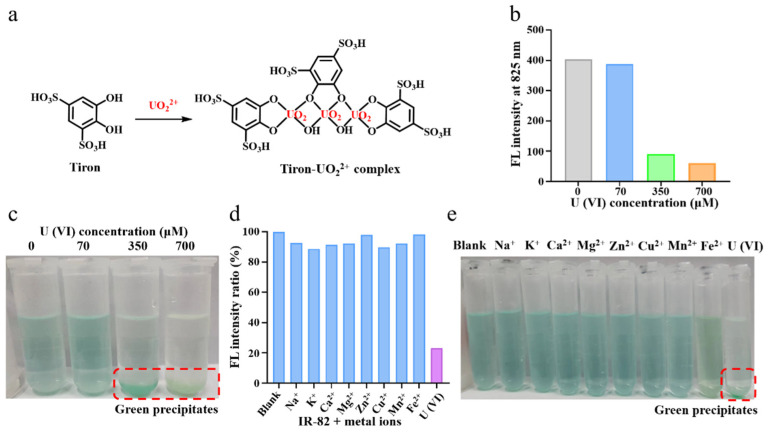
Chelation ability of IR-82 towards UO_2_^2+^. (**a**) The chelating mechanism of a catechol unit against UO_2_^2+^ based on the literature [29] (**b**) The fluorescence intensity of IR-82 after the addition of different concentrations of UO_2_^2+^. (**c**) The precipitates observed after chelation between IR-82 and high concentrations of UO_2_^2+^. (**d**,**e**) The selective chelation of IR-82 by evaluating the fluorescence intensity and precipitate generation in different metal ions.

A previous study shows that Tiron with a catechol unit can chelate UO_2_^2+^ to form stable complexes (Figure 1a) [29]. In order to evaluate the chelation ability of IR-82 towards UO_2_^2+^, the spectral characteristics of IR-82 were compared and analyzed before and after the addition of UO_2_^2+^. As shown in Figure S6, IR-82 exhibited two main UV absorption peaks at 720 nm and 798 nm, respectively, while the corresponding maximal fluorescence emission peak was at 825 nm in Tris-HCl buffer solution (Figure S7, Supplementary Materials). Therefore, a fluorescent intensity change at 825 nm was monitored to verify the chelation effect of IR-82 towards UO_2_^2+^. The results showed that the addition of UO_2_^2+^ led to a fluorescence intensity reduction when the UO_2_^2+^ concentration was up to 350 μM (Figure 1b and Figure S8, Supplementary Materials). In particular, a color fade and generated green precipitates were clearly observed by the naked eye in both groups with UO_2_^2+^ concentration of 350 μM and 700 μM, providing solid evidence of a chelation effect between IR-82 and UO_2_^2+^ (Figure 1c and Figure S8, Supplementary Materials). A molecular ionic peak at *m*/*z* 1110.5156 was detected in the mixture solution of IR-82 and UO_2_^2+^ by high-resolution mass spectrometry, further verifying the chelation effect by IR-82 and UO_2_^2+^ (Figure S9, Supplementary Materials). In addition, the specific chelation of IR-82 toward UO_2_^2+^ was explored by detecting the fluorescence intensity and visually observing green precipitates in the other different metal ions (Figure 1d,e). The results demonstrate that IR-82 can selectively chelate with UO_2_^2+^ rather than other metal ions, such as Na^+^, K^+^, Ca^2+^, Mg^2+^, Zn^2+^, Cu^2+^, Mn^2+^ and Fe^2+^. Overall, our findings in water solution above evidence that IR-82 has a good chelating ability towards UO_2_^2+^.

### 2.3. Protective Effect of IR-82 on HK-2 Cells after Uranium Exposure

**Figure 2:**
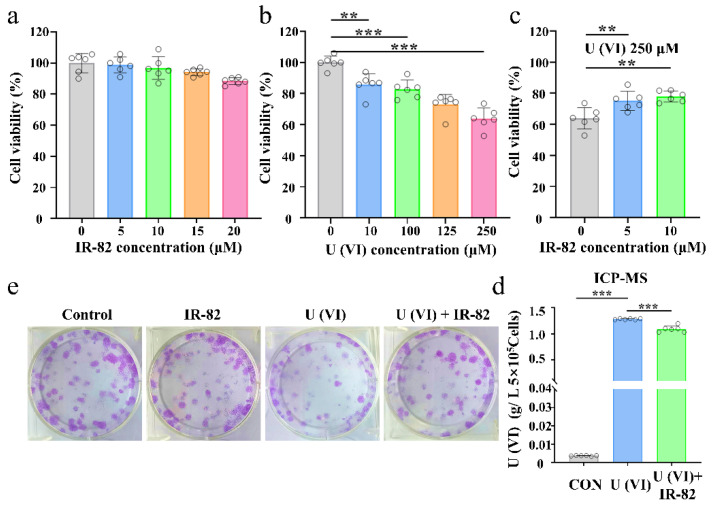
Protective effect of IR-82 on HK-2 cells after uranium exposure. (**a**) The cell viability was determined 24 h after treatment with different concentrations of IR-82. (**b**) The cell viability was determined 24 h after treatment with different concentrations of U(VI). (**c**) The cell viability was determined after being co-incubated with U(VI) (250 μM) for 24 h, following by indicated concentrations of IR-82 for another 24 h. (**d**) The uranium content in the cells was measured by ICP-MS. The cell viability was determined by CCK-8 assay. Data represent means ± SD of 6 separate experiments. ** *p* < 0.01, and *** *p* < 0.001. (**e**) The colony formation ability of IR-82 (10 μM) in the absence or presence of U(VI)-treated HK-2 cells (250 μM).

Based on the high affinity and selectivity of IR-82 for uranium, its toxicity was initially evaluated before other in vitro experiments were conducted on the cellular level. Considering that uranium is mainly deposited in renal proximal tubular epithelial (HK-2) cells to cause renal injury, the cytotoxicity of both IR-82 and U(VI) were assessed using HK-2 cells by CCK-8 kit assay. IR-82 exhibited no obvious toxicity to HK-2 cells within a concentration range of 0–20 μM (Figure 2a). The cytotoxicity of uranium assay was performed by adding different concentrations of U(VI) (0, 10, 100, 125 and 250 μM) to determine the appropriate concentration for the subsequent assays. The results show that uranium is significantly toxic to HK-2 cells at a concentration of 10 μM (Figure 2b). Cell viability was above 85.84% after incubation with 10 µM U(VI). In contrast, cell viability was reduced to 63.83% with 250 µM U(VI). The cell viability of HK-2 cells was determined after being co-treated with different concentrations of IR-82 (0, 5 and 10 μM) and U(VI) (250 μM). It indicated a significant detoxification effect of IR-82 (10 μM) against U(VI) (250 μM), and cell viability increased to 77.9% (Figure 2c). The cell uptake of U(VI) was quantitatively measured by ICP-MS, and the results demonstrated that U(VI) was significantly removed out of HK-2 cells (Figure 2d). The long-term protective effect of IR-82 against uranium was further examined using a clonogenic assay. Compared to the control group, the colony formation was inhibited in the uranium-exposed control group. In contrast, the addition of IR-82 significantly increased the cell survival rate and colony formation (Figure 2e), suggesting its effective detoxication against uranium. 

**Figure 3:**
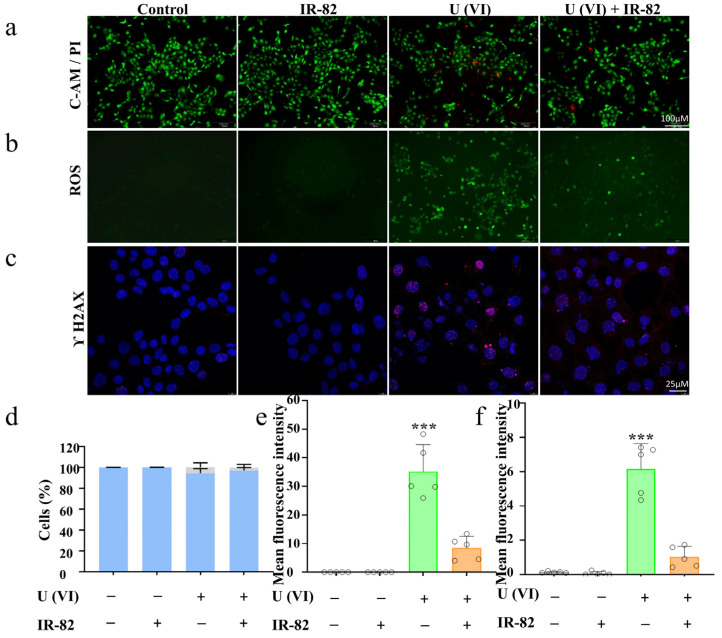
IR-82 amelioration of cell damage and DNA damage in uranium-exposed cells. HK-2 cells were incubated in the presence of IR-82 (10 μM) and U(VI) (250 μM). Effects of IR-82 on intracellular (**a**) viability/cytotoxicity, (**b**) ROS and (**c**) γ-H2AX levels after uranium treatment. ImageJ quantitation of (**d**) viability/cytotoxicity, (**e**) ROS and (**f**) γ-H2AX levels. Data represent means ± SD of 5 separate experiments. *** *p* < 0.001.

The protective effect of IR-82 against uranium toxicity was further confirmed by Calcein AM and propidium iodide (PI) co-staining tests (Figure 3a,d). The green Calcein AM dye and the red PI dye stain the live and dead cells, respectively. The results suggest that IR-82 could reduce the dead cells (red fluorescence) caused by uranium exposure. Uranium-exposed cells generate excessive reactive oxygen species (ROS), which eventually lead to cell apoptosis and necrosis. As shown in Figure 3b,e, uranium caused a sharp increase in reactive oxygen species (ROS) production, and IR-82 significantly reduced the ROS level induced by uranium exposure. Even aside from chemical toxicity [30,31,32,33], uranium radiation toxicity cannot be ignored. H2AX is a histone variant that is rapidly converted into the phosphorylation form as serine 139 (termed as γ-H2AX) and that mediates DNA damage in response to double-strand breaks (DSBs). The existing evidence has shown that DSB occurrence can be triggered by exposure to ultraviolet radiation, ionizing radiation and radiomimetic and ROS-induced heavy metals [34]. Therefore, γ-H2AX has been widely accepted as a “gold standard” to evaluate the extent of DSBs. As displayed in Figure 3c,f, both the intensity and number of γ-H2AX foci in the U(VI) group were apparently higher than those in any of the three groups, suggesting apparent DNA damage after uranium exposure. However, those of the U(VI)+IR-82 group were remarkably lower than the U(VI) group. These results indicate that IR-82 could serve as a potential anti-radiation agent to reduce ROS levels and DNA damage in cells.

### 2.4. Mitochondria-Targeted Accumulation and Function Protection 

**Figure 4:**
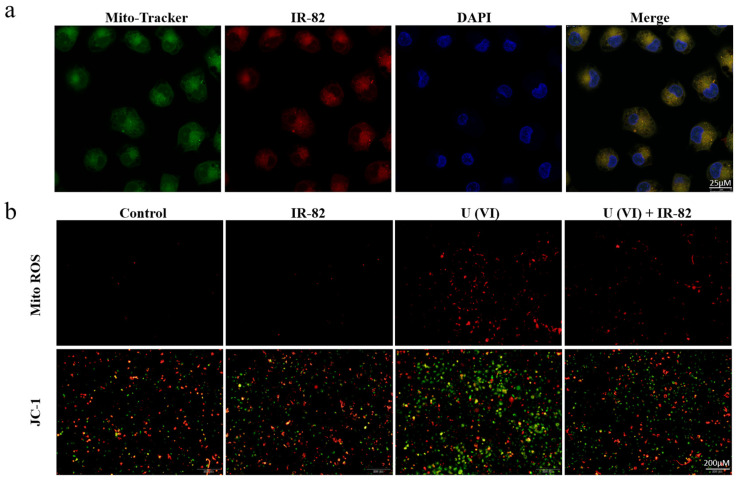
Mitochondria-targeted IR-82 for scavenging Mito-ROS and maintaining mitochodria membrane potential of HK-2 cells after uranium treatment. (**a**) Subcellular localization of IR-82 (red) was detected by a mitochondria-specific probe (Mito-Tracker, green). (**b**) Alterations in mitochondrial ROS (Mito-ROS, red) and mitochondrial membrane potential (JC-1) were detected after exposure to uranium U(VI) (250 μM) for 24 h, following by treatment with IR-82 (10 μM) for another 24 h.

The subcellular localization of IR-82 was investigated by using a commercial Mito-Tracker. The results in Figure 4a show that IR-82 can specifically accumulate in mitochondria because the green of the Mito-Tracker co-localized well with the red of IR-82. As mentioned above, ionizing radiation can induce mitochondrial ROS generation, mitochondria dysfunction and even cell apoptosis. We also examined the mitochondrial ROS-scavenging ability of IR-82 using a Mito-ROS red tracker. The results show that IR-82 can effectively reduce the Mito-ROS induced by U(VI) (Figure 4b). In a mitochondrial membrane potential assay, the non-U(VI) group showed no significant change in cell membrane potential, while a loss of mitochondrial membrane potential was significantly observed in the U(VI) group. The cell membrane potential increased significantly in the IR-82+U(VI) group, suggesting that IR-82 has a certain protective effect on uranium-induced mitochondrial membrane damage. 

### 2.5. Kidney Metabolism and Protective Effect of IR-82 in U(VI)-Exposed Mice 

**Figure 5:**
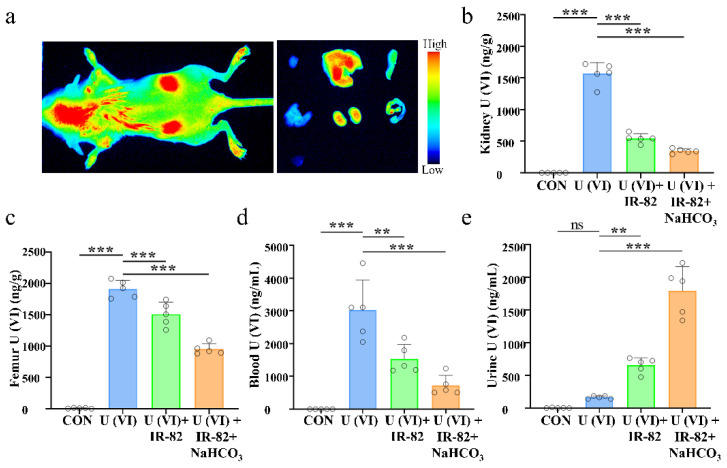
Kidney metabolism and uranium decorporation of IR-82 in mice. (**a**) In vivo NIR imaging properties of IR-82 at 6 h post-administration. Uranium content in kidney (**b**), femur (**c**), blood (**d**) and urine (**e**) were measured by ICP-MS. Results represent means ± SD of 5 separate experiments. ** *p* < 0.01, and *** *p* < 0.001, “ns” represents no significance, compared with uranium-treated cells.

We assessed the NIR fluorescence properties of IR-82 by imaging BALB/c mice. IR-82 gradually accumulated in the kidney regions, with a peak at 6 h postintravenous injection (Figure S10, Supplementary Materials). We also examined and compared the biodistribution of IR-82 in the major organs (heart, liver, spleen, lung, kidney, intestine) and muscle. Ex vivo imaging revealed that the kidneys exhibited stronger fluorescence signals compared with the other organs, verifying the preferential accumulation of IR-82 in the kidneys (Figure 5a). According to Figure 5b, the content of uranium in the kidneys of mice in the control group, U(VI) group, U(VI)+IR-82 group and U(VI)+IR-82+NaHCO_3_ group was 4.35 ± 1.98, 15,663.49 ± 1752.31, 5444.01 ± 757.55 and 3437.89 ± 371.91 ng·g^−1^, respectively. Compared to the U(VI) group, there were 65.24% and 21.09% reductions in the kidneys and humerus respectively, after IR-82 treatment (Figure 5b,c). More importantly, the uranium content in the kidneys decreased by 78.05% after combination use of IR-82 and NaHCO_3_, which was better than the 74.0% and 68.87% reported in the literature [3,12]. NaHCO_3_ has been well acknowledged as a representative of uranium decorporation agents. Uranium content in the blood decreased by 49.08% in the U(VI)+IR-82 group and 76.10% in the U(VI)+IR-82+NaHCO_3_ group (Figure 5d). In contrast, the uranium content in urine increased significantly in the U(VI)+IR-82 group and U(VI)+IR-82+NaHCO_3_ group (Figure 5e).

**Figure 6:**
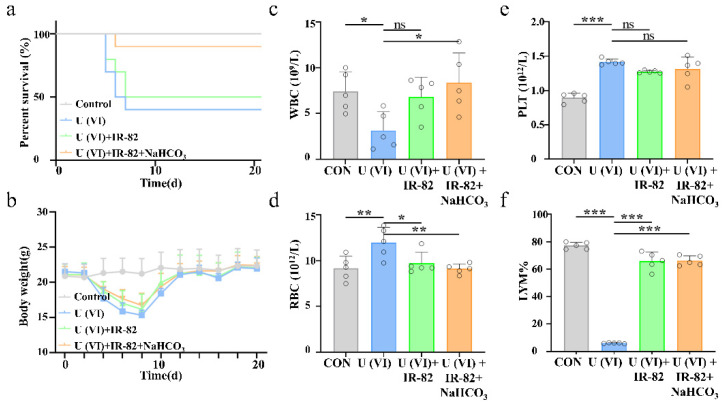
Effects of IR-82 on mouse survival, body weight and blood routine after uranium exposure. (**a**) Percent survival of mice in different groups. (**b**) Body weight of mice in different groups. The contents of WBC (**c**), RBC (**d**), PLT (**e**) and LYM% (**f**) in mice were measured by automated Konelab 20. Results represent means ± SD of 5 separate experiments. * *p* < 0.05, ** *p* < 0.01, *** *p* < 0.001, “ns” represents no significance, compared with uranium-treated cells.

After uranium exposure, the death of the mice occurred on day 5, 6, and 7 without any treatment. IR-82 protects against uranium exposure in mice as a uranium decorporation agent. The survival rate of the U(VI) group was 40%, that of the U(VI)+IR-82 group was 50% and that of the U(VI)+IR-82+NaHCO_3_ group was 90% (Figure 6a). IR-82 ameliorates the weight loss caused by U(VI) exposure in mice (Figure 6b). When whole blood was collected for routine blood test, IR-82 also improved WBC (White Blood Cell), RBC (Red Blood Cell), PLT (Platelet) and LYM% (Lymphocyte percentage) levels in mice three days after uranium exposure (Figure 6c–f).

**Figure 7:**
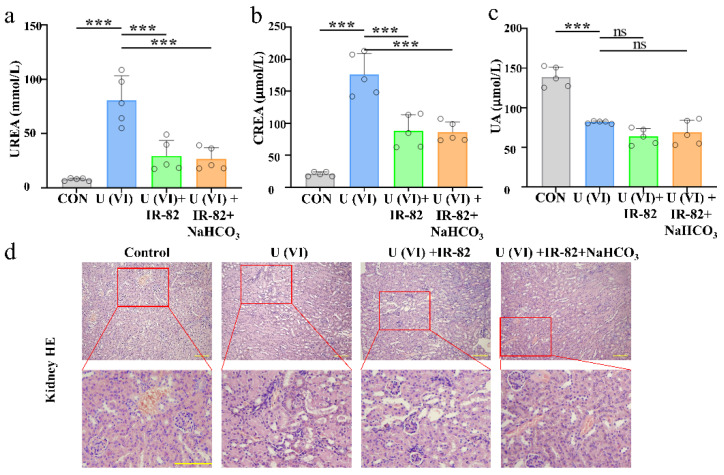
IR-82 improved the kidney functions of uranium-exposed mice. The blood biochemical indicators associated with kidney function indexes UREA (**a**), CREA (**b**) and UA (**c**) in mice were measured. (**d**) H&E staining of kidney tissues from mice after uranium exposure and indicated treatments. *** *p* < 0.001, “ns” represents no significance, compared with uranium-treated group.

The kidney is one of the body’s primary organs for detoxification, which makes the kidneys highly susceptible to uranium harm. Urea nitrogen (UREA), creatinine (CREA) and blood uric acid (UA) are blood biochemical indicators that can measure kidney functions. UREA and CREA are recognized as signs of glomerular filtration. As show in Figure 7a–c, the levels of the UREA and CREA indexes in the uranium-treated group were the highest among all groups, indicating renal damage in the mice model. In contrast, those parameters in the IR-82 treated group were much lower than the U(VI) group. It is therefore deduced that IR-82 can effectively relieve the renal damage induced by uranium (Figure 7a,b). Nevertheless, there was no significant change in UA in the IR-82-treated mice, compared to uranium-treated mice (Figure 7c). We analyzed renal tissue samples under a light microscope and observed that the extent of the injuries varied among the experimental groups. In the renal tissue of the U(VI) group (Figure 7d), we observed a large number of tube-shaped structures with hyaline degeneration in the renal tubular epithelial cells, cell vacuolization, abscission and necrosis and mononuclear cell infiltration in some interstitial tissues. Interestingly, these pathological injuries were attenuated in U(VI) + IR-82 group and U(VI) + IR-82 + NaHCO_3_ group. It is therefore deduced that IR-82 can effectively relieve the damage of uranium to the kidneys.

## 3. Materials and Methods

### 3.1. Material Preparation

All the chemicals in this work were commercially available and directly used without further purification. 3,4,5-trihydroxybenzoic acid, 6-bromohexan-1-ol, 2,3,3-trimethyl-3H-indole, 1,4-butanesultone and sodium acetate (NaOAc) were purchased from Aladdin Biochemical Technology (Aladdin, Shanghai, China). Sulfuric acid, n-hexane, ethyl acetate, acetonitrile, ethanol (EtOH), dichloromethane (DCM), methanol (MeOH), toluene and 1-butanol were obtained from Titan Scientific Co., Ltd. (Adamas, Shanghai, China). Radionuclide uranium fragments were provided by the China National Munitions Corporation (Beijing, China), composed of 99.25% uranium and 0.75% titanium by weight. Uranium fragments were dissolved in concentrated nitric acid, diluted in double-distilled water to 2 mg/mL, filtered and sterilized in a 0.22 μM filter unit (Millipore, Billerica, MA, USA) and stored at 4 °C for later use. Uranium was administered as uranyl nitrate, because uranyl ions (UO_2_^2+^), abbreviated as U(VI), are the most stable species of uranium in the environment and mammalian body fluids. 

HK-2 (human kidney 2) is a proximal tubular cell (PTC) line derived from normal kidneys. HK-2 cells (CL-0109) were purchased from the Committee on Type Culture Collection of the Chinese Academy of Sciences (CCTCC, Wuhan, China) and grown in Minimum Essential Medium (MEM, Procell, Wuhan, China) supplemented with 10% (*v*/*v*) fetal bovine serum (FBS, Hyclone, South Logan, UT, USA), 1% (*v*/*v*) penicillin and streptomycin (GIBCO, Grand Island, NY, USA). The cells were incubated in an atmosphere of 5% CO_2_ at 37 °C and propagated every other day.

### 3.2. Chemical Synthesis and Structure Characterization

#### 3.2.1. Synthesis of S1

The intermediate S1 was synthesized by an optimized method according to the previous literature [35]. To a 25 mL pressure bottle, 3,4,5-trihydroxybenzoic acid (2.35 g, 13.82 mmol), 6-bromohexan-1-ol (8.17 g, 42.85 mmol) and 0.6 mL concentrated sulfuric acid was added. The mixture was stirred at 120 °C for 30 min, then cooled to 100 °C, and it continued to be stirred for another 3 h. The reaction process was monitored by TLC (n-hexane/ethyl acetate = 1:1). When the reaction was completed, the solution was cooled to room temperature and diluted with 20 mL distilled water. The mixture solution was extracted with ethyl acetate (3 × 30 mL) and dried by anhydrous sodium sulfate. After filtering, the remainder was evaporated in a vacuum and subsequently purified by column chromatography on silica gel (n-hexane/ethyl acetate = 3:1) to give a colorless oil, 6-bromohexyl-3,4,5-trihydroxybenzoate S1 (2.76 g, 60% yield). ^1^H NMR (600 MHz, DMSO-d6)δ9.25 (s, 2H), 8.95 (s, 1H), 6.94 (s, 2H), 4.18 (t, J = 6.0 Hz, 2H), 3.34–3.32 (m, 6H), 2.49 (s, 2H), 2.17–2.13 (m, 2H) ppm.

#### 3.2.2. Synthesis of S2

A 100 mL round-bottom flask was charged with S1 (1.34 g, 4.02 mmol) and 2,3,3-trimethyl-3H-indole (0.96 g, 6.03 mmol) dissolved in 10 mL anhydrous acetonitrile. The mixture was heated to 110 °C and refluxed for 16 h under N_2_ protection. When the reaction was finished, the solution was cooled to room temperature and evaporated in a vacuum. The residue was purified by column chromatography on silica gel (DCM/MeOH = 25:1) to give a white solid S2 (1.03 g, 52% yield). ^1^H NMR (600 MHz, DMSO-*d*_6_) δ 9.18 (s, 2H), 8.90 (s, 1H), 7.92–7.91 (m, 1H), 7.78–7.77 (m, 1H), 7.56–7.55 (m, 2H), 6.88 (s, 2H), 4.39 (t, *J* = 7.8 Hz, 2H), 4.10 (t, *J* = 6.6 Hz, 2H), 2.78 (s, 3H), 1.82–1.77 (m, 2H), 1.64–1.58 (m, 2H), 1.47 (s, 6H), 1.44–1.37 (m, 4H) ppm.

#### 3.2.3. Synthesis of S3 and S4 

The 2,3,3-Trimethyl-1-(4-sulfonatobutyl)-3H-indolium S3 and the key intermediate semi-cyanine S4 were prepared according to our previously reported methods [26].

#### 3.2.4. Synthesis of IR-82

A 25 mL round-bottom flask was charged with S2 (113.4 mg, 0.25 mmol) and S4 (98.4 mg, 0.2 mmol). A 5 mL mixture solution of toluene/1-butanol (3.5/1.5) was added, and the reaction was heated to 120 °C and refluxed for 10 h under N_2_ protection. When the reaction was finished, the solution was cooled to room temperature and evaporated in a vacuum. The residue was purified by column chromatography on silica gel (DCM/MeOH = 35:1) to give a green solid, IR-82 (49 mg, 29% yield). ^1^H NMR (600 MHz, CD_3_OD) δ 8.40 (dd, *J* = 21.0, 13.8 Hz, 2H), 7.51 (dd, *J* = 12.0, 7.8 Hz, 2H), 7.44–7.38 (m, 3H), 7.31–7.25 (m, 3H), 7.05 (s, 2H), 6.32 (d, *J* = 14.4 Hz, 1H), 6.23 (d, *J* = 13.8 Hz, 1H), 4.23 (t, *J* = 6.0 Hz, 4H), 4.17 (t, *J* = 7.2, 2H), 2.90 (t, *J* = 7.2 Hz, 2H), 2.68–2.64 (m, 4H), 2.04–1.99 (m, 2H), 1.97–1.92 (m, 2H), 1.88–1.84 (m, 4H), 1.79–1.75 (m, 2H), 1.72 (d, *J* = 9.6 Hz, 12H), 1.59–1.54 (m, 4H) ppm. ^13^C NMR (151 MHz, CD_3_OD) δ 173.1, 172.2, 167.0, 149.6, 145.1, 144.5, 143.5, 142.2, 142.1, 141.3, 141.0, 138.3, 128.5, 128.5, 126.9, 126.5, 125.2, 124.9, 122.1, 120.2, 111.1, 110.7, 108.6, 101.4, 100.5, 64.1, 50.3, 49.3, 49.1, 43.7, 43.5, 28.2, 26.9, 26.9, 26.7, 25.9, 25.9, 25.8, 25.4, 22.2, 20.6 ppm. High-resolution mass spectrometry (HRMS) [M + H]^+^ calcd for C_47_H_56_ClN_2_O_8_S^+^, 843.3440; found, 843.3000. 

### 3.3. Determination of Chelating Ability towards UO_2_^2+^

To determine its chelating ability against UO_2_^2+^, IR-82 was dissolved in Tris-HCl buffer (0.02 M, pH 7.4) to a final concentration of 10 μM, and U(VI) was added to the above solution to a final concentration of 14 μM, 28 μM, 42 μM, 56 μM, 70 μM, 350 μM and 700 μM, respectively. Fluorescence emission spectra (IR-82: λ_ex_ = 805 nm) were detected after interacting for 12 h at room temperature. 

To determine the antijamming capability of IR-82 toward UO_2_^2+^, IR-82 was dissolved in Tris-HCl buffer (0.02 M, pH 7.4) to a final concentration of 10 μM, and different metal ions, including Na^+^, K^+^, Ca^2+^, Mg^2+^, Zn^2+^, Cu^2+^, Mn^2+^ and Fe^2+^, were added by replacing the uranyl ion to the above solution to a final concentration of 350 μM. Fluorescence emission spectra (λ_ex_ = 805 nm) were detected after interacting for 12 h at room temperature. 

### 3.4. In Vitro Experiments of IR-82 for U(VI) Detoxification

#### 3.4.1. Cytotoxicity and Viability Assay

HK-2 (5000 cells/well) were cultured with 100 μL medium in a 96-well plate for 24 h. The cytotoxicity assay was performed by adding different concentrations of IR-82 (0, 5, 10, 15, 20 μM) or U(VI) (0, 10, 100, 125, 250 μM) to determine the appropriate concentration for the subsequent assays [8,36]. After 24 h, the CCK-8 (MCE, Shanghai, China) solution (10 μL) was added to each well and incubated for 2 h at 37 °C. Finally, the optical density (OD) value at 450 nm was measured by a microplate reader (Thermo Multiskan GO, Waltham, MA USA). The survival viability was determined by the following formula: [OD (experimental group) − OD (blank group)]/[OD (control group) − OD (blank group)] × 100%.

The cell viability assay was cultured with 5000 cells in a 96-well plate. After 24 h, the culture medium was replaced (with U(VI) and IR-82) and then incubated for 24 h. Cell viability was detected by the Cell Counting Kit-8 as conducted according to the manufacturer’s protocol. And then for each experiment, the cells were exposed to U(VI) (250 μM) and treated with IR-82 (10 μM) diluted in complete medium.

#### 3.4.2. Cell Clonogenic Survival Assay

Exponentially growing HK-2 cells (500 cells/well) were seeded in 6-well plates and allowed to adhere overnight. The next day, the cells were exposed to U(VI) (250 μM) and treated with IR-82 (10 μM) diluted in MEM medium. The cells were cultured for 3 weeks and fixed with 4% paraformaldehyde (Biosharp, Hefei, China) for 10 min. Crystal violet staining solution (Beyotime, Shanghai, China) was used to stain the cells for 10 min after washing. Then, the colonies were washed and observed.

#### 3.4.3. Calcein and PI Assay

HK-2 cells (3 × 10^5^ cells/well) were seeded on 6-well plates and cultured in 1 mL MEM for 24 h. The cells were exposed to U(VI) (250 μM) and treated with IR-82 (10 μM) at 37 °C for 24 h. Then, the cells were washed with PBS (Boster, Pleasanton, CA, USA) and incubated in Calcein AM, PI (Beyotime, Shanghai, China) at 37 °C for 30 min. Finally, the cells were observed by fluorescence microscope, and the images were quantitatively analyzed.

#### 3.4.4. Determination of Intracellular ROS Production

For ROS determination, the cells were stained with DCFH-DA (reactive oxygen species assay kit, Beyotime, Shanghai, China). After entering the cell, DCFH-DA can be hydrolyzed by the esterase in the cell to generate DCFH. Intracellular reactive oxygen species can oxidize non-fluorescent DCFH to produce fluorescent DCF. The level of intracellular reactive oxygen species can be determined by measuring the fluorescence of DCF. HK-2 cells were seeded on 6-well plates at 3 × 10^5^ cells per well and cultured in 1 mL MEM for 24 h. The cells were exposed to U(VI) (250 μM) and treated with IR-82 (10 μM); after 24 h, the cells were incubated for 30 min at 37 °C with 10 μM DCFH-DA [37]. After the incorporation of the fluorescent probe, the cells were washed twice with PBS and observed by fluorescence microscope, and the images were quantitatively analyzed.

#### 3.4.5. Uranium Uptake Experiments in HK-2 Cells

Exponentially growing cells were exposed to 250 μM U(VI) and treated with 10 μM IR-82. The uranium-exposure-alone control was treated with uranium, and the untreated control was treated with saline. After 24 h, the cell cultures were counted with a cell counter and collected for centrifuge; the cells were lysed with concentrated nitric acid, and the U(VI) contents were analyzed by Inductively Coupled Plasma Mass Spectrometry (ICP-MS).

#### 3.4.6. Immunofluorescence

The cells were exposed to U(VI) (250 μM) and treated with IR-82 (10 μM) for 24 h, and fixed with aspirate liquid containing 4% formaldehyde for 15 min under room temperature. Then, the cells were washed with PBS, blocked with Blocking Buffer (Beyotime, Shanghai, China) for 60 min and incubated with phospho-H2A.X (Cell Signaling Technology, Danvers, MA, USA, Phospho-Histone H2A.X (Ser139) MousemAb, 80312S, 1:400 dilution) overnight at 4 °C. The cells were promptly washed with PBS and incubated with Anti-Mouse (Cell Signaling, Anti-Mouse IgG (H + L) F(ab′)2 Fragment, which was conjugated to Alexa Fluor^®^ 555 fluorescent, 4409, 1:1000 dilution) in the dark for 2 h at room temperature. The nuclei were counterstained with DAPI (MCE, Shanghai, China) in the dark for 10 min at room temperature. The cells were observed by confocal fluorescent microscope, and the images were quantitatively analyzed. Blue and red colors represented DAPI-stained nuclei and γ-H2AX foci, respectively.

#### 3.4.7. Mitochondrial Localization

A total of 2 × 10^5^ HK-2 cells were incubated in laser confocal dishes and cultured in a cell incubator for 24 h. After the incubation of IR-82 (10 µM) for 24 h, the cells were washed twice with PBS, 1 mL Mito-tracker green (250 nM, Thermo Fisher, Waltham, MA, USA) staining solution was added, and they were incubated for 30 min at 37 °C. The cells were washed twice with PBS, and 1 mL 4% paraformaldehyde was added to fix the cells for 10 min. The cells were washed twice with PBS, and 1 mL DAPI staining solution was added, and they were incubated for 10 min at 37 °C. The cells were washed twice again with PBS, and 1 mL 70% glycerol was added to seal the slice. The cells were observed by confocal fluorescent microscope.

#### 3.4.8. Determination of Mitochondrial ROS Production

The amount of ROS produced in mitochondria was measured using Mito-ROS Red CMXRos (Yeasen, Shanghai, China). HK-2 cells seeded (3 × 10^5^ cells/well) in 6-well plates overnight were treated with U(VI) (250 μM) and IR-82 (10 μM) for 24 h. Briefly, the treated cells were incubated with 10 μM Mito-ROS Red CMXRos probes at 37 °C for 30 min. Then, the cells were washed three times with serum-free medium and observed by fluorescence microscope.

#### 3.4.9. Microscopic Examination of Mitochondrial Membrane Potential

HK-2 cells were seeded on 6-well plates at 3 × 10^5^ cells per well and cultured in 1 mL MEM for 24 h. Next, the cells were exposed to U(VI) (250 μM) and treated with IR-82 (10 μM) for 24 h. The cells were incubated for 30 min at 37 °C in the dark with JC-1 dyeing working solution supplied in a mitochondrial membrane potential assay kit with JC-1 (Beyotime, Shanghai, China). At a high mitochondrial membrane potential, JC-1 aggregates in the matrix of mitochondria and forms a polymer (J-aggregates), which produces red fluorescence. When the mitochondrial membrane potential is low, JC-1 cannot aggregate in the matrix of mitochondria. At this time, JC-1 is a monomer and can produce a green fluorescence. After incubation, the cells were washed three times with a wash buffer supplied in the kit and observed by fluorescence microscope.

### 3.5. In Vivo Experimental for U(VI) Decorporation 

#### 3.5.1. Animal U(VI) Exposure

The uranium exposure models were divided into four groups: the control group, U(VI) group, U(VI)+IR-82 group and U(VI)+IR-82+NaHCO_3_ group. Each group was pretreated with or without 2.5 mg/kg IR-82 or 300 μL 5% NaHCO_3_ at the indicated concentrations for 1 h before the ip intraperitoneal injection of 2.5 mg/kg U(VI) to each mouse [12]. The survival of the mice was observed every day, and the weight of the mice was measured every other day.

#### 3.5.2. Experiments on the Effects of Uranium Exposure on the Organs of Mice

Three days after the ingestion of uranium, blood, serum and tissue samples were collected. Then, the blood routine and renal function were tested. Urea nitrogen (UREA), creatinine (CREA) and blood uric acid (UA) in serum were measured by an automatic biochemical analyzer. The mouse main organs were weighed or the liquid volumes were determined and then digested with 1 mL concentrated nitric acid at 90 °C using a microwave digester for 30 min. Each resulting specimen was diluted with ultrapure water, and the uranium concentration was determined using inductively coupled plasma mass spectrometry (ICP-MS, ThermoFisher, Ther-mICPOES7200, Waltham, MA, USA). Uranium levels were measured in a variety of tissues by ICP-MS to analyze the dynamic changes and distribution of uranium in the mice. Values are expressed as ng/g (or ng/mL) tissue. The preserved mouse viscera organ sections were cut with a microtome, stained with hematoxylin and eosin, and examined by light microscope. 

#### 3.5.3. IR-82 Accumulation and Metabolism in Kidney

We assessed the NIR fluorescence properties of IR-82 to survey accumulation and metabolism in the kidneys (Pearl Trilogy, Lincoln, NE, USA). IR-82 was injected into the tail vein to observe the accumulation of IR-82 in vivo and in vitro for 15 min, 2 h, 6 h, 24 h and 48 h. 

#### 3.5.4. Histological Observation

At the 7th day after the administration of uranium, the kidneys were dissected and fixed with 4% formaldehyde for 48 h, dehydrated and embedded in paraffin, then sliced into 4 μm sections. Hematoxylin and eosin (H&E, Beyotime, Shanghai, China) staining was used to observe the pathomorphological changes. The morphology of the glomerulus (normal or abnormal), tubules (normal or with alterations) and vessels were evaluated.

### 3.6. Statistical Analysis

The data in this work were reported as the mean ± SD of 3–6 replicate assays. Data were analyzed and plotted using GraphPad Prism 7 or OriginPro 8. Statistical analyses used one-way analysis of variance (ANOVA). *p*-values were considered to be statistically significant as * *p* < 0.05, ** *p* < 0.01 and *** *p* < 0.001.

## 4. Conclusions

In this work, a new uranium chelator, IR-82, is successfully synthesized and reported for attenuating uranium-induced nephrotoxicity. IR-82 is prepared based on mitochondria-targeted heptamethine indocyanine fluorescent small molecules and a further modification with GA. According to fluorescence intensity change and precipitates generation, including mass spectrometry, IR-82 displays a steady and specific chelation effect towards U(VI) in water solution. In uranium-treated HK-2 cells, IR-82 is demonstrated to accumulate in mitochondria and reduce mitochondrial ROS and intracellular uranium content, significantly improving cell viability. Water-soluble IR-82 tends to efficiently chelate the uranium deposited in the kidneys, promote the formation of U(VI)-IR-82 complexes in urine, and feasibly be excreted out through the kidneys. Thus, both the animal survival rate and kidney functions are found to be significantly improved in IR-82-treated groups. More interestingly, the combination use of NaHCO_3_ can further enhance the detoxification effect of IR-82. Collectively, the designed compound IR-82 shows a dose-dependent fluorescence change and steady chelation effect towards uranium exposure, indicating a potential application in both uranium detection and detoxification. Nevertheless, IR-82 also has limitations, such as a relatively low total yield of synthesis, difficulty in large-scale production and exhibiting toxicity at high concentrations, resulting in a narrow therapeutic window. Therefore, an optimized synthetic method or structural modification based on IR-82 is warranted in our future work to improve the potential of its practical application. In summary, our study may present a promising strategy and develop a new uranium chelator for effectively attenuating uranium-induced nephrotoxicity.

## Data Availability

Data are contained within the article.

## Supplementary Materials

The following supporting information can be downloaded at: https://www.mdpi.com/article/10.3390/ph17080995/s1, Figure S1: ^1^H NMR of compound S1; Figure S2: ^1^H NMR of compound S2; Figure S3: ^1^H NMR of IR-82; Figure S4: ^13^C NMR of IR-82; Figure S5: HRMS spectra of IR-82; Figure S6: UV absorption spectra of IR-82; Figure S7: Fluorescent emission spectra of IR-82; Figure S8: U(VI) selective chelation by IR-82 among different metal ions; Figure S9: HRMS spectra of IR-82 and UO_2_^2+^ mixture water solution; Figure S10: IR-82 metabolism in mice.
