# Supplementary material for: A Mitochondria-Targeted Heptamethine Indocyanine Small Molecular Chelator for Attenuating Uranium Nephrotoxicity
Source: Pharmaceuticals (Basel). 2024 Jul 27;17(8):995. doi: 10.3390/ph17080995 (PMC11357497; doi:10.3390/ph17080995)
Supplement: Supplementary file 1 [file pharmaceuticals-17-00995-s001.zip › pharmaceuticals-3107638-supplementary.pdf]

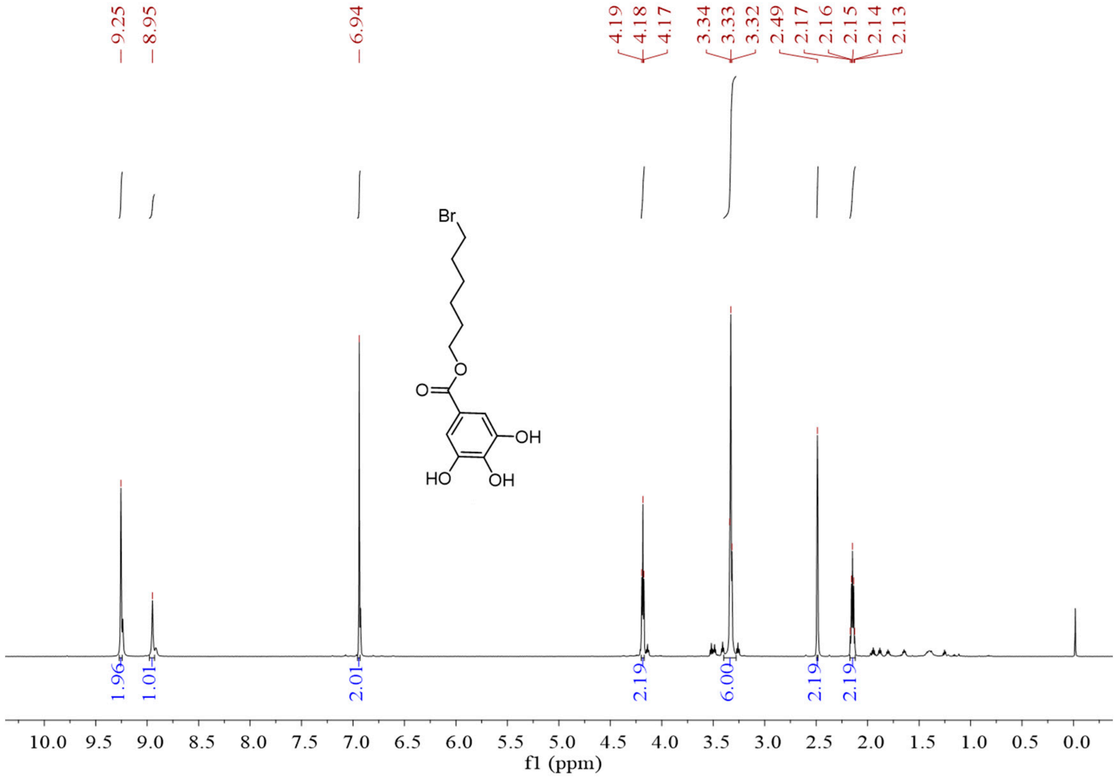

Figure S1. ^{1}H NMR spectra of S1.

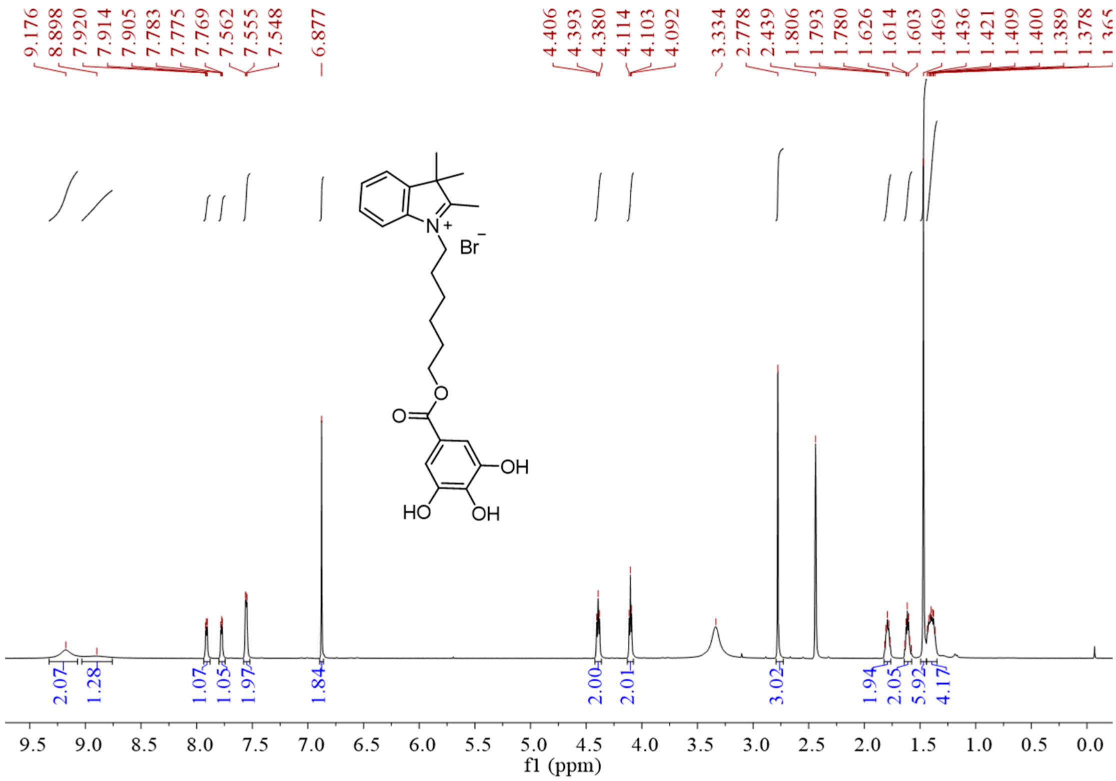

Figure S2. ^{1}H NMR spectra of key intermedates S2.

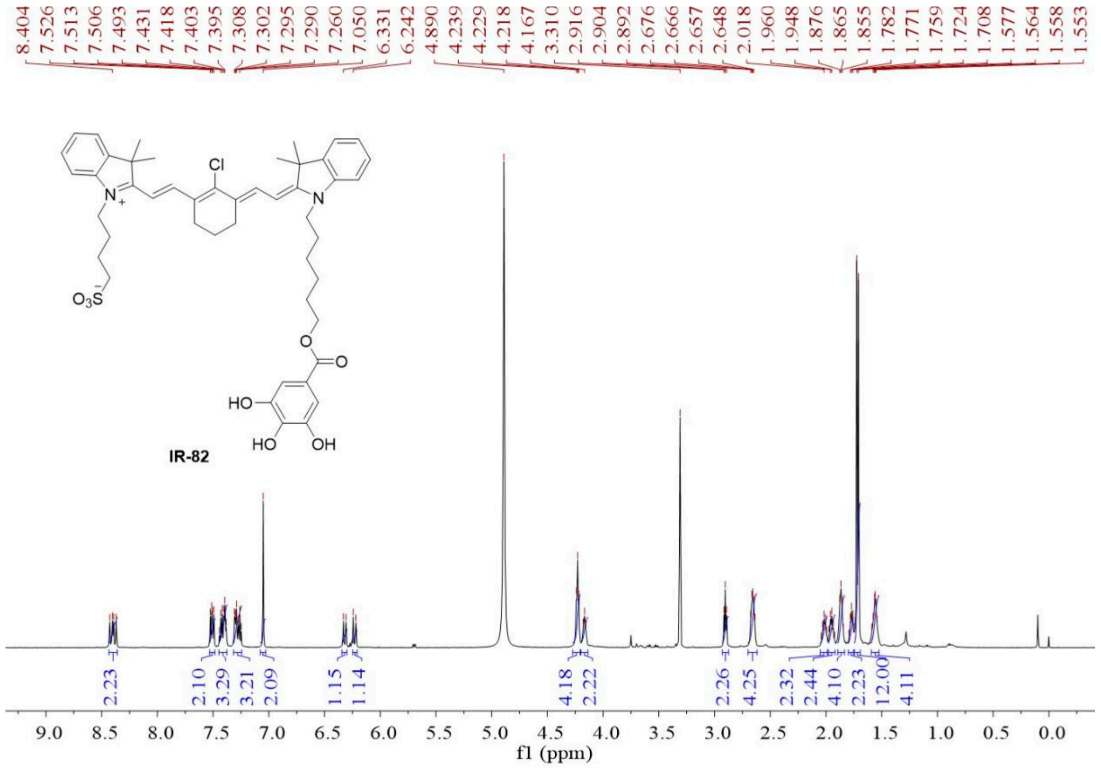

Figure S3. ^{1}H NMR spectra of IR-82.

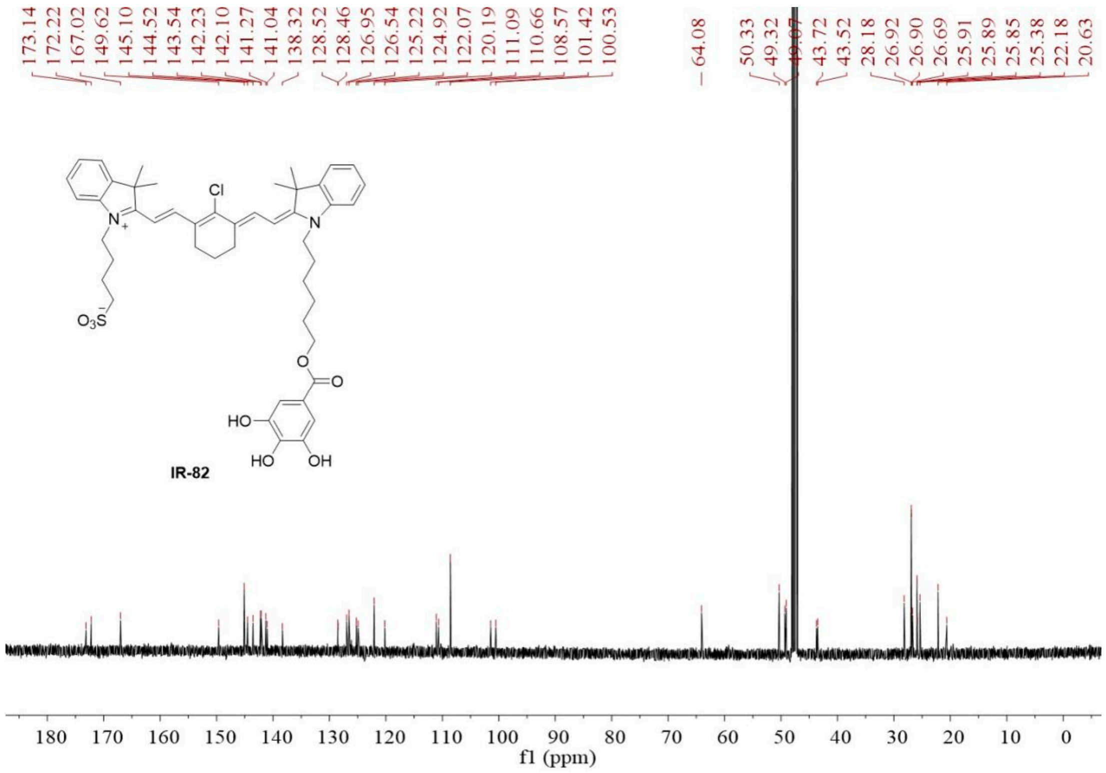

Figure S4. ^{13}C NMR spectra of IR-82.

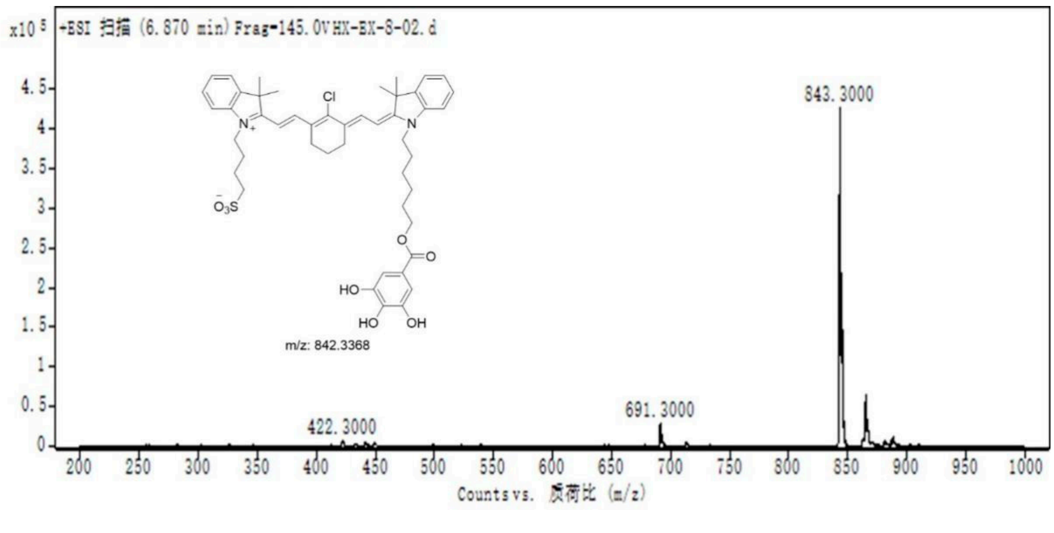

Figure S5. HRMS spectra of IR-82.

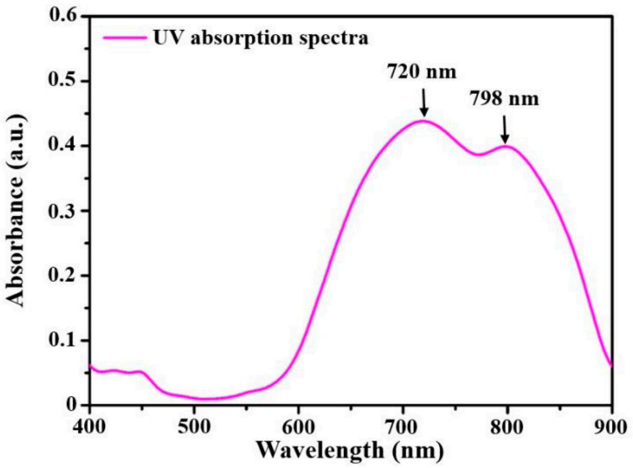

Figure S6. Ultraviolet absorption spectra of cyanine molecule IR-82 (10 μM) in 0.02 M Tris-HCl solution (pH = 7.4).

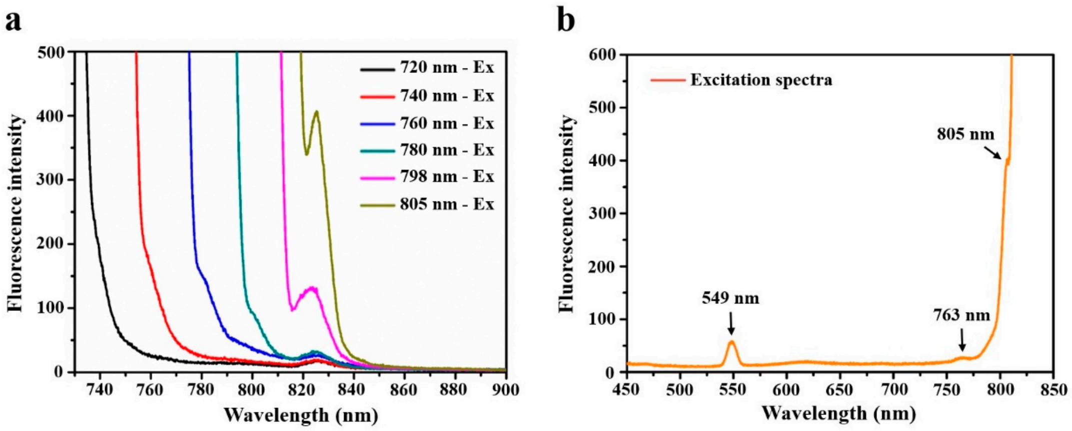

Figure S7. (a) Fluorescence emission spectra of IR-82 (10 μM) at different excitation wavelengths. (b) Fluorescence excitation spectra of IR-82 at 825 nm fluorescence emission. The maximum fluorescence excitation wavelength of cyanine molecule IR-82 is 805 nm, and the maximum fluorescence emission wavelength is 825 nm.

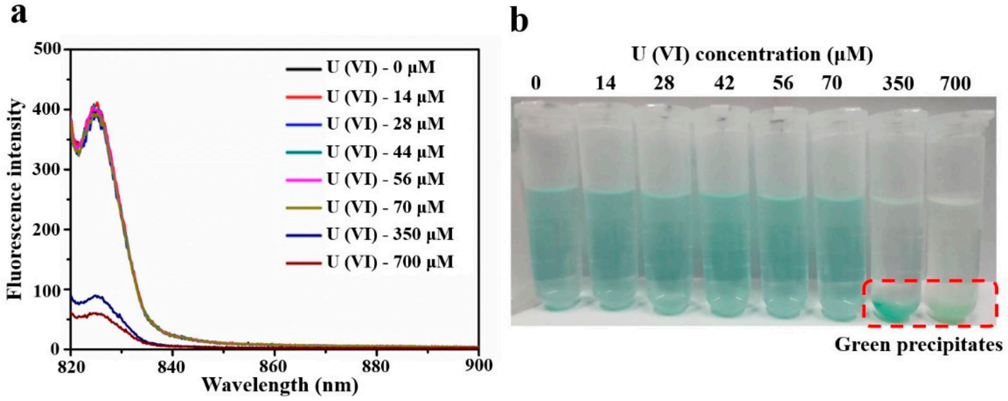

Figure S8. Fluorescence response of IR-82 (10 μM) toward different concentrations of UO_2^{2+} (a) and corresponding mixture solution (b). When the concentration of UO_2^{2+} increased to 350 μM and 700 μM, the generated green precipitates by IR-82 and UO_2^{2+} could be clearly observed by naked eyes.

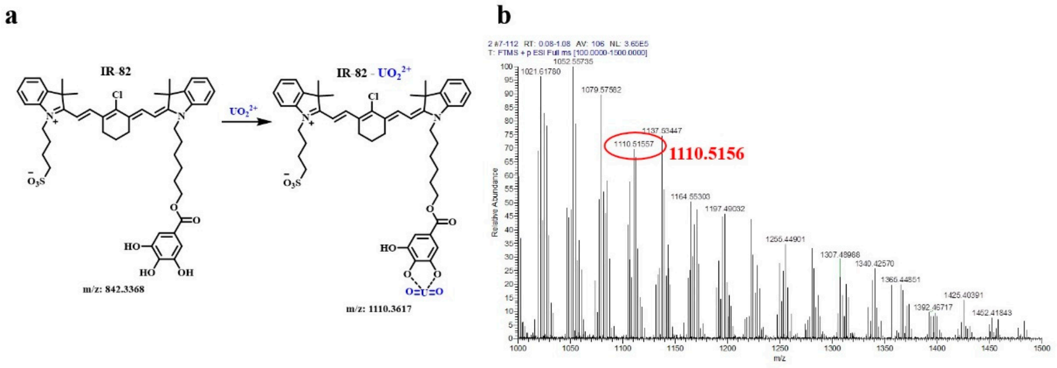

Figure S9. (a) The possible chelation form of IR-82 toward UO_2^{2+}. (b) HRMS spectra of IR-82 and UO_2^{2+} mixture solution.

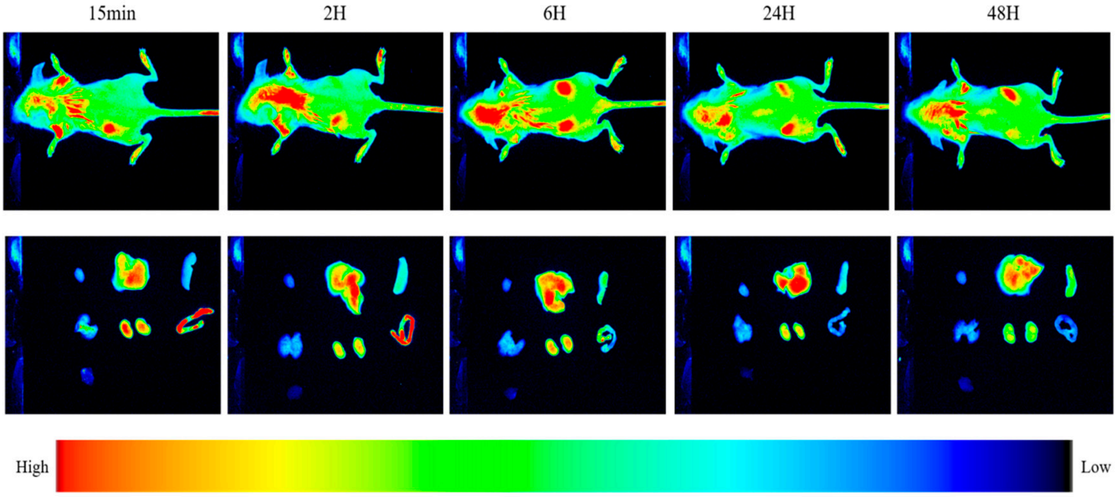

Figure S10. IR-82 metabolism in mice. (a) In vivo and ex vivo NIR fluorescence images of normal BALB/c mice taken at 15 min, 2 h, 6 h, 24 h and 48 h after IR-82 was administrated via i.p. injection.
